# Dietary Disruptors in Romania: Seasonality, Traditions, and the COVID-19 Pandemic

**DOI:** 10.3390/nu17010183

**Published:** 2025-01-03

**Authors:** Adrian Pană, Ștefan Strilciuc, Bogdan-Vasile Ileanu

**Affiliations:** 1 Center for Health Outcomes & Evaluation, Splaiul Unirii 45, 030126 Bucharest, Romania; adrian.pana@me.com (A.P.); bogdan.ileanu@csie.ase.ro (B.-V.I.); 2 Department of Genomics, MEDFUTURE Institute for Biomedical Research, Iuliu Hațieganu University of Medicine and Pharmacy, 400337 Cluj-Napoca, Romania; 3 RoNeuro Institute for Neurological Research and Diagnostic, 400364 Cluj-Napoca, Romania; 4 Department of Statistics and Econometrics, Bucharest University of Economic Studies, 010552 Bucharest, Romania

**Keywords:** dietary habits, energy intake, food choice, COVID-19, obesity, Romania

## Abstract

Background: The global rise in obesity has been significantly influenced by shifts in dietary habits that have been exacerbated by external factors such as the COVID-19 pandemic. This study aims to analyze the trends in Romanian dietary habits from 2015 to 2023, focusing on the impact of the COVID-19 pandemic and the role of socio-economic factors, seasonality, and cultural practices. Methods: For dietary habits, we used nationally representative data from the Romanian Household Budget Survey provided by the Romanian National Institute of Statistics. The survey includes 30,000 households annually. From the same provider, we downloaded data about potential drivers of food consumption, such as income, the consumer price index, and the unemployment rate. The analysis mixes descriptive statistics and panel data analysis. Among the main drivers, the econometric models include seasonality and regional factors, ensuring a comprehensive understanding of the changes in dietary behavior. Results: During the COVID-19 pandemic, daily calorie consumption increased to over 3000 calories per person, representing a 20% increase compared to the pre-pandemic period. Post-pandemic, food consumption remains elevated, averaging 2500–2600 calories per person daily. The pandemic also led to a shift in dietary composition, with significant changes. Thus, we mark an increase in fat (*p* < 0.001) and carbohydrate intake (*p* < 0.01) and a decrease in protein intake (*p* < 0.001). Beyond the presence of health disruptors, we confirm the significant impact of income (*p* < 0.001) and seasonality (*p* < 0.001). Other factors like unemployment, the consumer price index, and hidden regional factors have a minor role. Conclusions: The COVID-19 pandemic has had a lasting impact on Romanian dietary habits, reinforcing unhealthy eating patterns that were already prevalent. The sustained increase in calorie consumption, particularly of nutrient-poor, energy-dense foods, poses a significant public health challenge. The study also highlights significant seasonal variations, with a marked increase in food intake during the last quarter of the year, driven by cultural and religious traditions. These findings underscore the need for targeted public health interventions and policies that address economic factors and cultural and regional influences to promote healthier dietary behaviors in Romania.

## 1. Introduction

The global prevalence of overweight and obesity has reached alarming levels, with the World Health Organization (WHO) reporting in 2022 that nearly 60% of adults and one-third of children are now classified as overweight or obese, in WHO European regions [1]. This increase in unhealthy weight has become a critical public health issue, driven by a complex interplay between dietary habits, lifestyle factors, and socio-economic conditions. Over the last decade, the rise in obesity has been linked to increased consumption of energy-dense foods, particularly those rich in refined sugars, fats, and processed meats [2]. Modern sedentary lifestyles have exacerbated these dietary trends, which are characterized by decreased physical activity and prolonged periods of inactivity.

The situation mirrors global trends in Romania but is further compounded by specific local factors, such as traditional dietary patterns and socio-economic disparities. Romania has one of the highest rates of overweight and alcohol consumption in Europe [3,4]. The country’s dietary habits are heavily influenced by dietary cultural practices, religious traditions, and seasonal dietary patterns, contributing to significant fluctuations in food intake throughout the year.

The COVID-19 pandemic introduced unprecedented challenges that further altered dietary behaviors on a global scale. In response to the pandemic, many countries, including Romania, implemented strict measures to curb the spread of the virus. These measures included lockdowns, social distancing, and the closure of various cultural, social, and economic activities, many closely associated with food consumption. Mobility restrictions within and between countries disrupted daily routines, leading to a sharp decline in physical activity as sports facilities closed and many jobs transitioned to remote work [5]. The pandemic also caused significant disruptions in supply chains, affecting the availability and variety of food products and influencing consumption patterns [6]. These disruptions, coupled with heightened levels of stress, anxiety, and depression, led to a shift toward unhealthy eating habits characterized by increased consumption of comfort foods high in fats, sugars, and carbohydrates.

While the literature provides ample evidence of changes in dietary habits during the COVID-19 pandemic, the findings are mixed. Some studies report positive nutritional changes, such as an increase in the consumption of fruits and vegetables, as individuals seek to boost their immunity [7,8,9]. Other studies present a more nuanced picture, indicating positive and negative dietary shifts or no significant changes [10,11]. However, a substantial body of research suggests a general trend toward unhealthy eating behaviors during the pandemic, with a marked increase in energy-dense, nutrient-poor foods, including sugary snacks, processed meats, and fast food [12,13,14,15,16,17,18].

In Romania, the impact of the COVID-19 pandemic on dietary habits still needs to be explored, particularly in long-standing cultural practices and seasonal nutritional patterns. Given the country’s high prevalence of overweight and other diet-related health issues, it is crucial to understand how the pandemic may have exacerbated these trends. This study aims to fill this gap by analyzing changes in dietary habits in Romania from 2015 to 2023, specifically focusing on the effects of the COVID-19 pandemic and the subsequent recovery period. By utilizing nationally representative data from the Household Budget Survey (HBS) provided by the Romanian National Institute of Statistics (NIS), this study offers a comprehensive overview of how Romanian dietary patterns have evolved and the socio-economic, seasonal, and regional factors that have influenced these changes.

This study is particularly timely given the ongoing public health challenges associated with obesity and diet-related chronic diseases. The findings are expected to contribute to the development of targeted public health interventions and policies aimed at improving dietary habits and reducing the burden of obesity in Romania. Furthermore, the insights gained from this study may have broader implications for understanding the long-term impact of global crises, such as the COVID-19 pandemic, on dietary behaviors and public health.

## 2. Materials and Methods

### 2.1. Study Population and Data Collection

The master dataset, formed by a multidimensional structure, includes the average monthly food consumption per person in the eight Romanian NUTS 2 regions for 18 main foods or groups of food products in 33 trimesters between 2015Q1 and 2023Q1. The Romanian National Institute of Statistics (NIS) provides such data in the online database Tempo Online [19,20,21], and the values are available in physical units, such as liters for beverages and kilograms for solid food. According to the Romanian National Institute of Statistics metadata, the Household Budget Survey (HBS) is a national representative sample applied to over 3000 independent dwellings/month, or over 30,000 independent units/year, from all Romanian counties [20,21]. It follows the Eurostat regulations [22]. The classification of consumed products and the questionnaire used to collect data are available on the Eurostat methodology website [22].

Local data are available on calories only for the leading group of products. Therefore, we used data from the United States Department of Agriculture [23], and we converted all average values from physical units/person/month to calories/person/day to improve the comparability among product categories and regions. Furthermore, standardization of quantities allows us to sum up the values for broader categories such as carbohydrates, proteins, or fats.

### 2.2. The Set of Variables Used in the Data Analysis

Beyond the daily food consumption used as the main variable in our inquiry, we collected information about monthly gross income [24]. This variable is reported in Romanian local currency (RON) per household. Further, we added data about the monthly consumer price index, *CPI* [25], with reference to December 2014, and the unemployment rate, *UR* [26].

The *CPI* is available for almost every one of the 18 product categories at the national level. Therefore, it is assumed that the same will happen for each region. UR and income are available for each Romanian region but do not vary by product class. Additionally, we computed geometrical means to determine the monthly mean *CPI* in a particular trimester.

### 2.3. Data Analysis

The current approach commences from the unanimously accepted theory of Keynes [8], which considers income the primary driver of consumption. In the nearly nine decades of the available theory of consumption, many studies, including those carried out by the European Central Bank [27], Serletis [28], Kearney [29], and Salo et al. [30], have investigated subjects related to drivers of consumption from different perspectives. Because the current objective is to evaluate the impact of the crucial disruptor COVID-19, we border the basic model of Keynes with other components, such as the consumer price index (*CPI*), unemployment level, or seasonality.

Initially, we applied descriptive statistics methods to characterize the state of monthly consumption within the analyzed period. We also provided a general picture of the covariates in Appendix A.

The time series points to the dynamic behavior and possible sub-annual oscillations. Then, in line with other works like that of Mora et al. [17], for each class and subclass of products, we specified two types of panel models:

(a) The static fixed effects panel model (FE) has the following form:

$${lncal}_{k,i,t}=\beta_{0i}+\beta_{1}{lnCPI}_{k,t}+\beta_{2}{lnIncome}_{i,t}+\beta_{3}{UR}_{i,t}\;\;\;\;\;\;\;\;\;\;\;\;\;\;\;\;\;\;\;\;\;\;\;\;\;\;\;\;\;\;\;\;\;\;\;\;\;\;\;\;\;\;\;+\sum_{s=1}^{3}d_{s}X_{s}+c_{1}COVID19+c_{2}pCOVID19+\varepsilon_{k,i,t}$$

(b) The dynamic panel models have the following form:
  $${lncal}_{k,i,t}=\sum_{l=1}^{l\leq 4}\alpha_{l}{AR}_{i,t-l}+\sum_{l=0}^{l\leq 4}\gamma_{l}{lnCPI}_{k,t-l}+\sum_{l=0}^{l\leq 4}\lambda_{l}{lnIncome}_{i,t-l}\;\;\;\;\;\;\;\;\;\;\;\;\;\;\;\;\;\;\;\;\;\;\;\;\;\;\;\;\;\;\;\;\;\;\;\;\;\;\;\;\;\;\;\;\;\;\;\;\;+\sum_{l=0}^{l\leq 4}\delta_{l}{UR}_{i,t-l}+\sum_{s=1}^{3}d_{s}X_{s}+c_{1}COVID19+c_{2}pCOVID19\;\;+u_{k,i,t}$$

where $ln{cal}_{k,i,t}$ represents the natural logarithm of daily calories per capita consumption, lnCPI and *lnIncome* are the natural logarithm of the *CPI* and monthly *Income* respectively. *UR* is the unemployment rate, and $X_{s}$ represents seasonal dummy variables, with winter as the reference category. *AR* is the autoregressive component of consumption. Five indexes account for the class and subclass of products (*k*), region (*i*), season (*s*), time lag (*l*), and time (*t*). *COVID*-19 accounts for 2020. *pCOVID*-19 measures the post-COVID-19 impacts and covers the interval of 2021–2023. The reference period for these two variables is defined as 2015:Q1–2019:Q4. In each model, $\varepsilon_{k,i,t}$ and $u_{k,i,t}$ capture the influence of unknown factors. They are assumed to be Gaussian, independent, and identically distributed. The ordinary least squares (OLS) method estimates the static model parameters. In contrast, the generalized method of moments (GMM) is applied to estimate the parameters of the dynamic models.

We applied several statistical tests, including the HEGY panel unit root test [31], the panel Durbin–Watson test (pDW) for residual autocorrelation, and Fisher and R-squared statistics, to assess the validity of the OLS model assumptions. The Sargan–Hansen (S-H) test validated the instruments used in the GMM models [32]. A *p*-value of 0.05 was used as the threshold for statistical significance. All computations and econometric analyses were conducted using Microsoft Excel (2018) [33] and R-Studio (R Core Team 2024) [34]. Further details about technical results can be consulted in Appendix A.

## 3. Results

### 3.1. Main Aspects Related to Food Consumption

**Figure 1:**
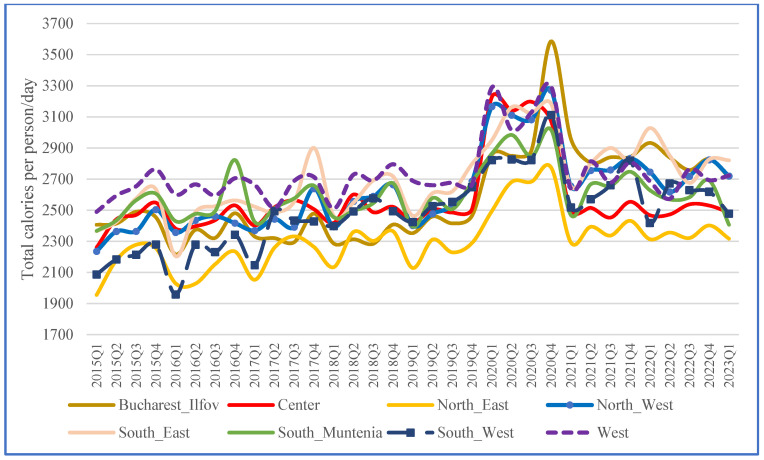
Trends of total energy intake by NUTS 2 region.

In the first available year, 2015, Romania’s daily average energy intake was approximately 2400 calories per person. This level remained relatively stable until 2019 when the mean daily value rose to 2505 calories. However, during the COVID-19 pandemic, daily food intake increased to over 3000 calories per person. By 2021–2023, consumption had decreased to around 2500–2600 calories per day, varying across seasons and regions. In the North-East and South-East regions, intake gradually increased, peaking in the last quarter of 2020. Conversely, in the West, North-West, and Central regions, consumption spiked early in 2020 before declining through Q1 2023 (Figure 1).

The Bucharest–Ilfov (BIF) region, Romania’s most developed area, experienced the most significant changes. By 2021–2023, calorie consumption in most regions had returned to pre-pandemic levels, with notable exceptions in the South-East (SE) and South-West (SW) regions. In the SE region, average consumption remained slightly higher than in the preceding five years. In contrast, the SW region exhibited a pronounced overall increase, with consumption rising from nearly 2100 calories per day to over 3300 calories during the peak of the COVID-19 pandemic and stabilizing above 2500 calories per day by the end of the study period.

The analysis of major macronutrient groups at the national level between 2015 and 2019 revealed that carbohydrates made up 70–75% of daily intake. Regional dietary patterns showed that in the West (W), North-West (NW), North-East (NE), and Bucharest–Ilfov (BIF) regions, the proportion of carbohydrates was lower.

**Figure 2:**
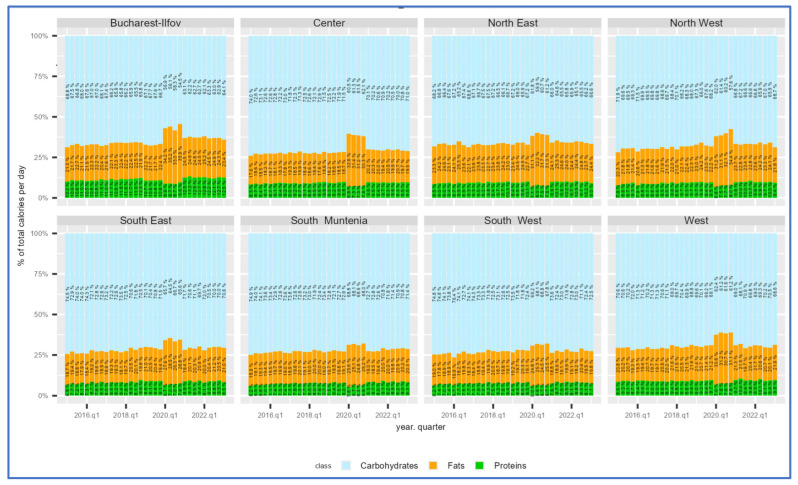
Share of calorie consumption by main class of goods, quarter, and region.

In contrast, in the other regions, it converged toward 75% (Figure 2). Protein consumption generally remained below 10% of daily intake, except in the Bucharest–Ilfov region, which reached 11% before the COVID-19 pandemic.

**Figure 3:**
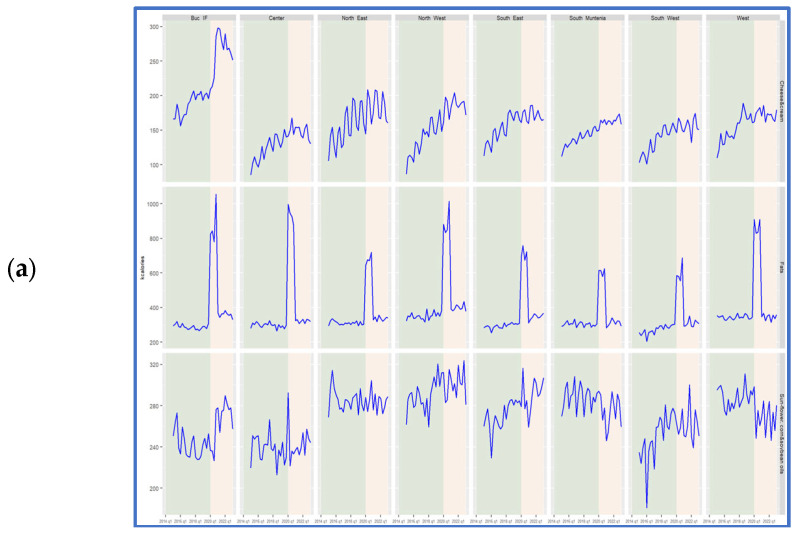

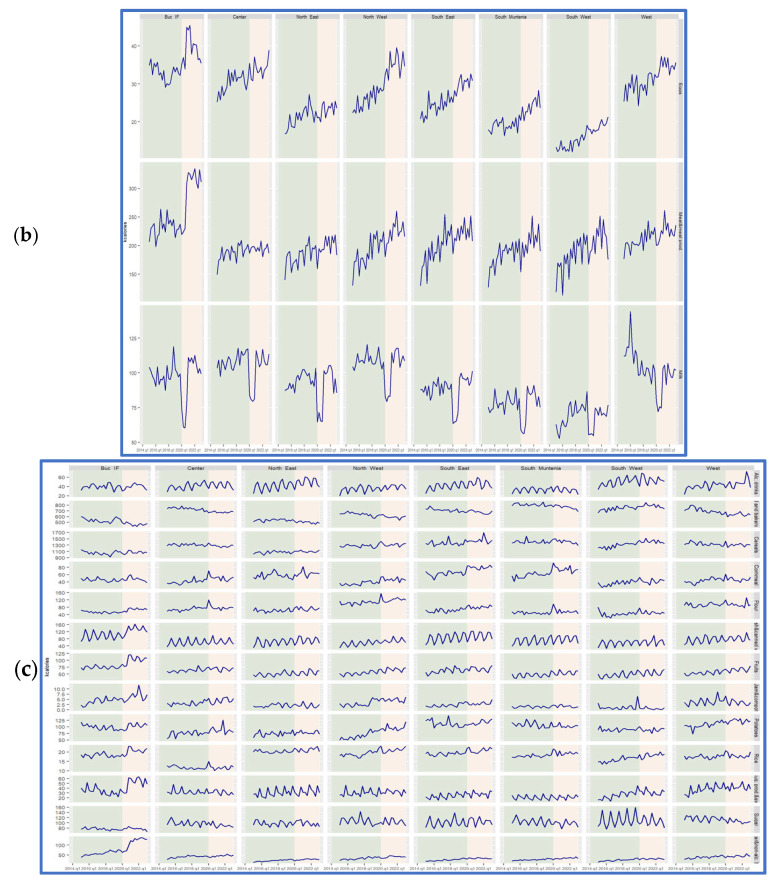
Quarterly trends by NUTS 2 regions of daily energy intake from fats (**a**), proteins (**b**) and carbohydrates (**c**).

Fats accounted for the remaining 20% of daily intake. Across all regions and throughout 2020, the share of fat intake increased significantly, with an increase ranging from 5 to 15 percentage points (p.p.) compared to the average of the previous five years. The most minor change occurred in the South-West (SW) region, with an increase of 5 p.p., while the most significant rise of nearly 15 p.p. was observed in the Bucharest–Ilfov area. Consequently, the share of proteins and carbohydrates decreased across all regions. The study further examined changes in macronutrient consumption by core categories—fats, carbohydrates, and proteins (Figure 3).

All fat categories exhibit clear seasonal variations with notable heterogeneity, particularly in the case of oils. The cheese and cream categories demonstrate a discernible trend during the period of 2015–2019, transitioning to a stationary pattern between 2020 and 2023. A pronounced anomaly was observed in the Bucharest–Ilfov region, where calories derived from cheese and cream significantly increased during the COVID-19 pandemic.

Fat consumption experienced a significant disruption during the pandemic, with mean caloric intake reaching 1000 kcal per person daily. This represented a quadrupling of fat-derived calories in the Bucharest–Ilfov and North-West regions, compared to a doubling in the North-East and South-Muntenia regions. Notably, fat consumption began to recover in 2021, returning to pre-pandemic levels by 2019.

Protein consumption also displayed strong seasonal variations, with higher heterogeneity observed in meat and processed meat consumption. Egg and meat consumption followed a general upward trend over the study period, whereas milk consumption showed no discernible increase or decrease. Despite its stability, milk consumption experienced a notable decline in 2020, affecting all eight Romanian regions, with the sharpest decrease observed in the Bucharest–Ilfov region and the smallest impact in the South-West region. This decline coincided with the lockdown period, after which milk consumption gradually recovered to 2019 levels by the second trimester of 2020.

COVID-19 also triggered anomalies in egg and meat consumption. Notable spikes in both categories were recorded in the Bucharest–Ilfov region. While egg consumption decreased post-restriction, meat and processed meat consumption remained elevated throughout the post-pandemic period. In the North-West region, egg consumption increased to nearly 35 kcal per person per day in 2021, reflecting a 50% increase compared to 2019 levels.

Carbohydrates, the largest group of products analyzed, displayed the least sensitivity to anomalies. Seasonal trends were evident, particularly in the consumption of alcoholic beverages, vegetables, fruits, and sugar. Unlike fats and proteins, carbohydrates showed no abrupt spikes during the study period. However, some notable changes emerged starting in 2020.

In the Bucharest–Ilfov region, fresh and canned vegetable consumption increased, which was accompanied by a rise in caloric intake from jams, compotes, and non-alcoholic beverages. Smaller, transient shocks in wheat flour consumption were observed in the Center, North-West, and South-Muntenia regions. Although less conspicuous in a broad analysis, these regional changes underscore the importance of examining consumption patterns with greater granularity.

### 3.2. Econometric Modeling and Main Outcomes

We observed a moderate positive and statistically significant correlation (*p* < 0.001) between the CPI and income, while the relationship between the UR and CPI was weakly linear. The econometric tests discussed in the methods section produced mixed results. Nonetheless, the stationarity of food consumption, income, and UR was generally accepted. Residual autocorrelation and the validity of GMM instruments led us to place greater confidence in the panel GMM results over the fixed effects (FE) model. Except for a few instances, there were no substantial discrepancies between the outcomes of the two modeling approaches (Table 1).

The CPI and UR occasionally impacted food consumption. The analysis confirmed that income is a crucial driver of consumption, which is consistent with the Keynesian theory for most goods. Additionally, the fixed effects models revealed minimal disparities among Romanian regions, suggesting that specific regional factors had a limited influence on food consumption patterns during the period studied.

The outcomes presented in Table 1 highlight various shifts in food demand. Cereal consumption typically increased by 1–5% during the cold season, and during the pandemic, this rise reached 2–3%, a trend that continued beyond 2020. Similarly, bread and bakery products exhibited seasonal variation, with no significant change attributable to the pandemic. In contrast, cornmeal and flour demand increased by 15–17% in the last quarter due to seasonality, and the COVID-19 period further boosted this demand by 20%. Water and non-alcoholic beverage consumption increased by 3–5% in the warmer months compared to winter. Despite Romania’s ample supply of local fruits, demand for fruits was 17–20% lower during peak availability compared to periods of scarcity. The data also indicate that the use of rice, potatoes, sweets, and sugar products decreased in the warmer seasons, and milk consumption exhibited only a modest seasonal rise of 3–5% during the colder months.

Viral shocks had minimal impact on most of these products, except for rice, which experienced a notable shift. In line with findings from France [16] and Spain [17], sugar demand increased by 5–7% during the pandemic. Among the 18 product categories analyzed, fats recorded the most dramatic increase, with demand rising by 60–90% during the pandemic’s peak compared to regular periods. Conversely, milk demand increased 30% during the pandemic’s stress wave before returning to its usual trend.

Regarding macronutrient intake, carbohydrates slightly increased by 2–3%, fats experienced a substantial rise of 30–40%, and protein demand dropped by nearly 10%. The final quarter of the year has an average growth of 5–10% in carbohydrate, protein, and fat consumption compared to earlier quarters, leading to a 38% increase in total calorie intake by year-end relative to July–September and a 2–5% increase compared to the first half of the year. Overall, COVID-19 led to a nearly 10% rise in total calorie demand compared to the pre-pandemic period. However, this elevated pattern did not persist into the post-pandemic period.

## 4. Discussion

The outcomes of this study provide a comprehensive analysis of the changes in Romanian dietary habits from 2015 to 2023, with a particular focus on the impact of the COVID-19 pandemic and the subsequent recovery period. The findings underscore the significant role of income, seasonality, and socio-economic factors in shaping food consumption patterns, which is consistent with the Keynesian consumption theory [28]. Additionally, in line with other results [35,36], this study highlights the marked influence of cultural and religious traditions on dietary behaviors, particularly during the last quarter of the year when significant Orthodox Christian holidays lead to increased consumption of high-calorie foods [35,37].

The COVID-19 pandemic substantially disrupted daily life, resulting in short-term and long-term dietary habit changes. The initial phase of the pandemic points to a dramatic increase in calorie intake, with daily consumption exceeding 3000 calories per person during the crisis. This rise was driven by several factors, including heightened stress levels, reduced physical activity due to lockdown measures, and changes in food availability and purchasing behaviors. The increase in the consumption of comfort foods rich in fats and sugars is consistent with global trends observed during the pandemic, where many individuals turned to energy-dense foods as a coping mechanism [2,6,35].

Our analysis further reveals significant regional disparities in how these dietary changes manifested. For example, the Bucharest–Ilfov region, Romania’s most developed area, exhibited the most pronounced shifts in consumption patterns. Calorie intake rapidly increased during the early months of the pandemic, followed by a gradual return to pre-pandemic levels. In contrast, the South-East and South-West regions experienced more sustained increases in calorie consumption, suggesting that socio-economic factors and regional differences in food accessibility and cultural practices may have contributed to these divergent trends.

The pandemic also intensified unhealthy dietary patterns, particularly the overconsumption of carbohydrates and fats, constituting most of the daily caloric intake [9]. While protein consumption decreased during the pandemic, likely due to supply chain disruptions and consumer preference shifts, the overall increase in calorie consumption suggests a move toward more energy-dense but nutritionally poor diets [27]. This shift is concerning, given the already high prevalence of obesity in Romania, and suggests that the pandemic may have exacerbated the risk of diet-related chronic diseases in the population [7].

Despite the return to more typical consumption patterns in the post-pandemic period, with daily caloric intake decreasing to around 2500–2600 calories per person, the lingering effects of the pandemic on dietary habits remain evident [5]. The continued preference for high-calorie foods, particularly during culturally significant periods, indicates that the pandemic may have had a lasting impact on Romanian food culture, reinforcing unhealthy eating behaviors that were already prevalent [2].

Moreover, the findings highlight the importance of considering the broader socio-economic and cultural context when analyzing dietary habits. While income remains a critical determinant of food consumption, as evidenced by our study’s strong correlation between income and calorie intake, other factors such as seasonality, cultural traditions, and regional disparities also play significant roles. These findings suggest that public health interventions aimed at improving dietary habits in Romania must take a multifaceted approach, addressing not only economic factors but also cultural and regional influences [38].

## 5. Conclusions

Our analysis reveals several critical insights into the trends of Romanian dietary habits from 2015 to 2023, particularly emphasizing the effects of the COVID-19 pandemic. The findings confirm that income, seasonality, and cultural factors are key drivers of food consumption patterns in Romania. The pandemic exacerbated unhealthy dietary behaviors, leading to a significant increase in the consumption of high-calorie, nutrient-poor foods, particularly during periods of cultural and religious significance.

The COVID-19 pandemic serves as a stark reminder of the vulnerability of dietary habits to external shocks, such as global health crises. The increased calorie intake during the pandemic and the continued preference for high-calorie foods post-pandemic suggest that Romania faces significant challenges in addressing diet-related health issues [12]. The pandemic’s impact on dietary habits has likely contributed to the growing prevalence of obesity and other chronic diseases, further straining an already overburdened healthcare system [4].

To mitigate the long-term effects of the pandemic on public health, it is essential to develop targeted interventions that address the specific dietary challenges faced by different regions and socio-economic groups in Romania. These interventions should prioritize improving access to healthy foods, particularly in regions that have shown more sustained increases in calorie consumption, and promoting cultural shifts toward healthier eating behaviors [18]. Public health campaigns should also focus on reducing the consumption of energy-dense, nutrient-poor foods, especially during periods of cultural and religious significance [15].

Furthermore, the findings underscore the need for comprehensive policies that address the broader determinants of dietary habits, including income inequality, food accessibility, and cultural practices. Such policies should promote healthier eating habits across all population segments, with particular attention to vulnerable groups at greater risk of diet-related health issues [16].

In conclusion, while the COVID-19 pandemic appears to have been a temporary anomaly in terms of its immediate impact on dietary habits, its long-term effects on public health in Romania are likely to be profound. Addressing these challenges will require coordinated efforts at the national and regional levels, involving public health authorities and community stakeholders, to promote healthier dietary behaviors and reduce the burden of diet-related diseases in the post-pandemic era [38].

## Figures and Tables

**Table 1:** Outcomes of primary regression analysis across model iterations.

**Model**	**Category**	**Method**	**lncal**				**lnIPC**				**UR**			**lnIncome**			**Trimester**			**Regions**								**Cov19**	**pCov19**	**Validation**		
			**AR1**	**AR2**	**AR3**	**AR4**	**t**	**t-1**	**t-2**	**t-3**	**t**	**t-1**	**t-2**	**t**	**t-1**	**t-2**	**Jan–Mar.**	**Apr–Jun.**	**Jul–Sept**	**BIF**	**C**	**NE**	**NW**	**SE**	**S**	**SW**	**W**			***R* ^2^**	**pDW**	**S-H**
1	Cereals	FE					−0.07				−0.001			0.02			−0.02	**−0.03**	**−0.04**	6.8	7.0	6.9	7.0	7.1	7.1	7.1	7.1	0.03	0.03	0.2	***	
2		GMM	**0.41**	0.16	0.22	0.21	−0.28	0.52	−0.52	0.25	0.002	−0.01	0.005	0.08	−0.13	0.05	**−0.03**	**−0.04**	**−0.05**									0.02	0.01			*p* = 0.99
3	Water& non-alcoholic beverages	FE					x				−0.02			**0.57**			−0.06	0.21	0.04	−0.6	−0.92	−1.4	−1.1	−1.2	−1.2	−1.3	−1.0	−0.08	0.01	0.8	***	
4		GMM	**0.50**	0.19	0.15	**0.16**	x	x	x	x	0.001	−0.01	0.01	**0.34**	**−0.34**	−0.004	−0.04	**0.06**	0.03									−0.07	−0.03			*p* = 0.99
5	Bread & bakery prod.	FE					−0.04				−0.003			**−0.07**			−0.03	−0.02	−0.02	6.9	7.2	6.9	7.1	7.2	7.3	7.3	7.2	−0.01	−0.02	0.4	***	
6		GMM	**0.54**	**0.20**	0.08	**0.20**	−0.32	0.30	0.20	−0.11	−0.01	0.01	−0.003	0.04	−0.04	−0.01	**−0.03**	−0.02	−0.02									−0.0003	0.0001			*p* = 0.99
7	Cornmeal	FE					−0.08				−0.01			**0.22**			0.04	−0.04	−0.04	1.9	2.0	2.3	1.8	2.5	2.4	1.9	2.0	**0.14**	0.04	0.4	***	
8		GMM	**0.38**	**0.27**	0.11	0.17	−0.51	0.13	1.7	−1.6	0.01	−0.03	0.03	0.20	−0.18	0.004	**0.07**	−0.04	−0.07									**0.10**	0.03			*p* = 0.99
9	Flour	FE					0.06				−0.01			0.05			−0.02	−0.04	**−0.15**	3.7	4.0	3.9	4.4	4.0	3.7	3.5	4.2	**0.20**	0.11	0.4	***	
10		GMM	**0.34**	0.28	0.14	0.15	−0.60	0.53	1.5	**−2.3**	−0.02	0.004	0.01	0.20	−0.38	0.23	−0.02	−0.07	**−0.18**									**0.13**	**0.16**			*p* = 0.99
11	Fresh & cannerd vegetables	FE					−0.28				0.01			**0.43**			**−0.45**	**−0.16**	**0.14**	1.1	0.7	0.89	0.7	1.2	0.91	0.7	1.0	0.02	0.03	0.8	0.7	
12		GMM	0.12	0.09	0.04	**0.68**	−0.05	−0.50	0.51	0.01	−0.002	−0.01	0.01	0.02	−0.23	0.03	−0.08	0.07	0.13									0.03	−0.005			*p* = 0.99
13	Fruits	FE					0.02				−0.01			**0.24**			−0.01	**−0.21**	**−0.23**	2.5	2.3	2.1	2.2	2.3	2.1	2.1	2.2	0.04	0.03	0.7	***	
14		GMM	**0.35**	0.03	0.15	**0.37**	−0.26	0.25	0.11	−0.15	0.01	−0.01	0.004	0.02	−0.09	0.14	−0.02	−0.14	**−0.17**									0.01	−0.02			*p* = 0.99
15	Jam & compot	FE					−1.1				−0.03			0.67			0.35	−0.04	−0.38	−4.2	−4.2	−4.6	−4.3	−4.4	−4.9	−5.6	−4.2	0.05	−0.05	0.3	***	
16		GMM	**0.25**	−0.02	0.39	**0.25**	−8.8	14.6	−5.5	0.72	−0.05	0.02	−0.01	0.18	−0.37	0.24	**0.30**	0.10	−0.27									−0.13	−0.15			*p* = 0.99
17	Rice	FE					0.12				−0.01			0.11			0.003	−0.04	−0.04	2.8	2.3	2.9	2.9	2.9	2.8	2.7	2.7	**0.06**	0.04	0.4	***	
18		GMM	**0.55**	0.16	0.15	0.12	−0.24	0.23	0.62	−0.78	−0.01	−0.003	0.01	0.04	−0.13	0.10	−0.01	**−0.06**	**−0.05**									0.04	0.03			*p* = 0.99
19	Potatoes	FE					−0.07				0.04			**0.17**			**−0.09**	−0.06	**−0.07**	3.2	3.0	3.0	3.0	3.4	3.3	3.2	3.4	0.03	0.02	0.2	***	
20		GMM	0.25	0.09	**0.30**	**0.26**	0.09	−0.13	−0.01	0.14	0.001	−0.01	0.01	**0.32**	**−0.33**	0.07	−0.11	−0.05	−0.05									0.01	−0.03			*p* = 0.99
21	Sugar products & sweets	FE					−0.26				−0.01			**0.20**			**−0.24**	**−0.31**	**−0.49**	2.2	2.1	2.1	2.0	1.9	1.7	2.0	2.3	0.03	0.07	0.6	***	
22		GMM	**0.40**	0.18	0.17	0.18	−0.50	−0.03	1.4	−1.1	−0.01	0.03	−0.02	0.20	−0.29	0.15	**−0.34**	**−0.35**	**−0.49**									−0.01	−0.03			*p* = 0.99
23	Sugar	FE					−0.04				−0.003			−0.08			**−0.11**	0.01	**0.11**	5.1	5.3	5.3	5.3	5.3	5.3	5.4	5.4	0.05	0.02	0.5	0.05	
24		GMM	**0.23**	−0.001	**0.22**	**0.54**	−0.10	0.12	−0.16	0.26	−0.01	0.01	0.01	0.22	−0.12	−0.99	**−0.06**	−0.01	0.02									**0.08**	0.01			*p* = 0.99
25	Meat & meat products	FE					−0.30				−0.01			**0.32**			**−0.17**	−0.04	−0.09	2.9	2.7	2.8	2.7	2.8	2.7	2.8	2.8	−0.05	0.05	0.7	***	
26		GMM	**0.36**	**0.25**	−0.08	**0.39**	**−1.8**	0.40	**2.3**	−0.73	−0.001	−0.01	0.01	0.17	−0.22	0.11	**−0.10**	0.02	−0.02									−0.04	−0.003			*p* = 0.99
27	Milk	FE					**−0.22**				−0.01			**0.10**			0.01	−0.03	**−0.06**	3.8	4.0	3.8	3.9	3.8	3.7	3.6	3.9	**−0.33**	0.02	0.8	***	
28		GMM	**0.36**	0.07	0.08	**0.26**	**−1.6**	0.69	0.67	0.48	−0.01	0.003	0.001	−0.007	−0.11	**0.27**	−0.03	−0.04	**−0.06**									**−0.30**	−0.002			*p* = 0.99
29	Eggs	FE					−0.15				−0.01			**0.26**			−0.04	−0.04	**−0.13**	1.3	1.3	1.0	1.2	1.2	1.0	0.7	1.3	0.05	**0.11**	0.7	***	
30		GMM	**0.31**	**0.30**	**0.19**	0.17	**−0.17**	0.13	0.02	0.05	−0.01	0.01	0.002	0.07	−0.05	0.002	−0.05	−0.05	**−0.13**									0.03	−0.01			*p* = 0.99
31	Cheese & cream	FE					**−0.29**				−0.01			**0.49**			−0.02	**0.07**	**0.07**	1.1	0.8	1.2	0.94	1.1	1.0	1.0	1.0	0.01	0.01	0.8	***	
32		GMM	**0.51**	−0.14	**0.34**	0.24	−0.46	0.55	0.51	−0.90	−0.004	−0.003	0.01	0.10	−0.06	−0.006	0.00	0.05	0.03									0.02	0.005			*p* = 0.99
33	Fats	FE					−0.02				0.01			0.08			**−0.05**	−0.04	**−0.05**	5.1	5.1	5.1	5.2	5.1	5.0	5.0	5.2	**0.87**	0.06	0.9	***	
34		GMM	**0.35**	−0.08	0.08	**0.08**	−1.9	3.4	−2.2	0.37	0.01	−0.02	0.02	0.44	−0.06	0.01	−0.02	0.01	−0.03									**0.61**	−0.02			*p* = 0.99
35	Sun-flower, corn, soybean oils	FE					0.01				−0.01			0.01			−0.03	−0.03	−0.02	5.5	5.4	5.6	5.6	5.6	5.6	5.5	5.6	0.01	0.01	0.1	***	
36		GMM	**0.31**	**0.21**	**0.24**	0.21	−0.19	0.31	0.16	−0.46	−0.01	−0.001	0.01	0.20	−0.22	0.04	−0.03	**−0.04**	**−0.03**									−0.003	0.02			*p* = 0.99
37	Total carbohydrates	FE									−0.01			0.03			**−0.05**	**−0.04**	**−0.03**	7.1	7.2	7.1	7.2	7.3	7.3	7.2	7.3	**0.03**	0.03	0.4	***	
38		GMM	**0.40**	0.13	0.20	**0.27**					−0.01	0.001	0.01	0.13	**−0.18**	0.05	**−0.06**	**−0.04**	**−0.04**									**0.02**	−0.002			*p* = 0.99
39	Total proteins	FE									−0.01			**0.21**			**−0.11**	−0.03	**−0.08**	4.2	4.2	4.1	4.2	4.2	4.1	4.0	4.2	**−0.12**	0.01	0.7	***	
40		GMM	**0.50**	**0.27**	−0.06	0.16					−0.001	−0.001	0.01	**0.16**	−0.23	0.16	**−0.12**	−0.02	**−0.06**									**−0.09**	**−0.05**			*p* = 0.99
41	Total fats	FE									0.01			**0.21**			**−0.04**	−0.01	−0.01	4.5	4.4	4.5	4.6	4.5	4.4	4.4	4.5	**0.64**	0.02	0.9	***	
42		GMM	**0.29**	0.02	0.05	**0.17**					−0.01	0.001	**0.02**	**0.48**	−0.07	−0.07	−0.02	0.02	−0.01									**0.46**	**−0.14**			*p* = 0.99
43	Total food	FE									−0.002			**0.09**			**−0.06**	**−0.03**	**−0.03**	7.1	7.1	7.0	7.1	7.2	7.1	7.1	7.2	**0.17**	0.02	0.8	***	
44		GMM	**0.40**	0.14	0.18	**0.28**					−0.01	−0.001	0.01	0.20	**−0.17**	−0.02	**−0.06**	**−0.03**	**−0.04**									**0.12**	−0.02			*p* = 0.99
		**−**	Significant at *p* < 0.001				**−**	Significant at *p* < 0.01				**−**	Significant at *p* < 0.055			*** serial correlation significant at *p* < 0.001																
		+					+					+																				

**Figure A1:**
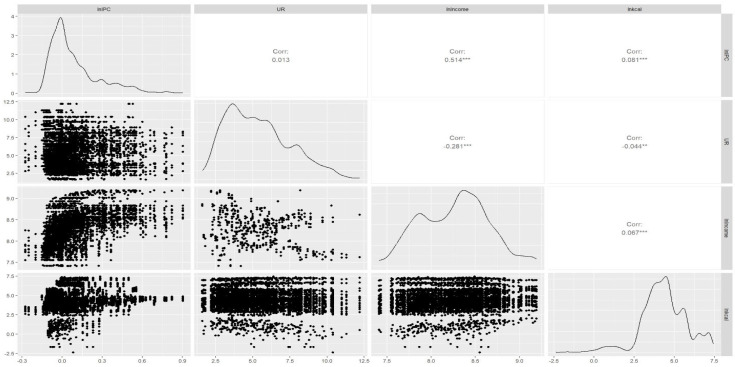
Descriptive statistics and correlations among consumption and its drivers.

## Data Availability

Data are available upon reasonable request from the corresponding author.

## Appendix A. Technical Details and Results of the Econometric Modelling

The static fixed effects panel model (FE) has the following form:

$${lncal}_{k,i,t}=\beta_{0i}+\beta_{1}{lnCPI}_{k,t}+\beta_{2}{lnIncome}_{i,t}+\beta_{3}{UR}_{i,t}+\sum_{s=1}^{3}d_{s}X_{s}+c_{1}COVID19+c_{2}pCOVID19+\varepsilon_{k,i,t}(a)$$

The dynamic panel models have the following form:

$${lncal}_{k,i,t}=\sum_{l=1}^{l\leq 4}\alpha_{l}{AR}_{i,t-l}+\sum_{l=0}^{l\leq 4}\beta_{l}{lnCPI}_{k,t-l}+\sum_{l=0}^{l\leq 4}\lambda_{l}{lnIncome}_{i,t-l}+\sum_{l=0}^{l\leq 4}\gamma_{l}{UR}_{i,t-l}++\sum_{s=1}^{3}d_{s}X_{s}+c_{1}COVID19\;\;+c_{2}pCOVID19+u_{k,i,t\;}(a)$$

where ${cal}_{k,i,t}$ represents the monthly consumption/person in calories, CPI is the consumption price index, *UR* is the unemployment rate, $X_{s}$ is a seasonal dummy variable with reference categories of winter, and *AR* is the autoregressive consumption component. Five indices, *k* = 1 to 19, *i* = 1 to 8, *s* = 1 to 3, *l* = 1 to max = 4, and *t* = 1 to 33, account for the class and subclass of products, region, season, time lag, and time, respectively. *COVID*-19 has a binary form with a value of 1 for all four seasons of 2020, and *pCOVID*-19 is a post-COVID-19 dummy variable with a value of 1 for all available seasons starting with the first quarter of 2021 (2021:Q1) to 2023:Q1. The reference period for *COVID*-19 and *pCOVID*-19 was pre-COVID-19, defined from the first quarter of 2015 to 2019:Q4. In both models, the $\varepsilon_{k,i,t}$ and $u_{k,i,t}$ variables are the disturbances, and they capture the influence of unknown factors. They are assumed to be normal, independent, and identically distributed.

Models from the (a) category also try to capture fixed effects such as religion, urbanization degree, or specific cultural propensity for some products, which are mainly linked to regional characteristics. Several assumptions, such as stationarity, exogeneity of covariates, and time-serial and cross-sectional independence of residuals, must be made to discuss the quantitative results of the models further. Accordingly, specific tests such as the seasonal HEGY panel unit root [31], Durbin–Watson statistics, and R-squared statistics were computed and analyzed to test this hypothesis. Then, the ordinary least squares (OLS) method was applied for parameter estimation.

The second category of models, (b), considers a probable invalidation of exogeneity and non-autocorrelation and homoskedasticity hypotheses from (a). The (b) class models then use a general method of moments (GMM) estimation. In addition, we captured via autoregressive (*AR*) components a potentially stable long-term consumption model based on an intrinsic cause associated with lifestyle factors that cannot be individualized with the current data.

We present only the most relevant among multiple sets of estimated equations, with various lag lengths, *l* and one-step vs. two-step estimations. The Sargan–Hansen (S-H) test, described by Arellano [32], was applied for the validity of instruments and accompanies the results of each model. When the series has the unit root and a high serial autocorrelation is present in the FE models, we considered that the dynamic panel with GMM outperforms the static analysis. A *p*-value less than 0.05 marks the cut-off for statistical significance for each test. Microsoft Excel [33] and R-Studio [34] were used to perform all the computational aspects and graphical representation.

Figure A1 was performed in R-studio, using GGaly library [39]. Following Emerson and colleagues [39] suggestion, * denotes significance at *p* < 0.05. Therefore, ** and *** flag significance at *p* < 0.01 and *p* < 0.001 respectively.

According to Hylleberg et al. [31] and following Otero et al. [40], the HEGY test has the following equation:

$${\Delta_{4}y}_{it}={miu}_{it}+{pi}_{1i}{y1}_{it-1}+{pi}_{2i}{y2}_{it-1}+{pi}_{3i}{y3}_{it-2}+{pi}_{4i}{y3}_{it-1}+\;\sum_{j=1}^{\max lag}\;\varphi_{ij}{\Delta_{4}y}_{i,t-j}+\varepsilon_{it},$$

where ${miu}_{it}=\alpha_{i}+\beta_{i}t+\sum_{s=1}^{3}\gamma_{is}D_{st}$, with $D_{st}$ = 1 in quarters 1…3, $y1=\left(1+L+L^{2}+L^{3}\right)y_{t}$, $y2=-\left(1-L+L^{2}-L^{3}\right)y_{t},\;y3=-\left(1-L^{2}\right)y_{t}$ and $\Delta_{4}=y_{t}-y_{t-4}$. The authors propose the following hypotheses:

- H0: ${pi}_{1}$ = 0; the unit root is present.
- H1: *pi*_2_ < 0; no unit root.
- H034: *pi*_3_ = *pi*_4_ = 0; the seasonal unit root is present.
- H134: at least *pi*_3_ or *pi*_4_ < 0; no seasonal unit root.

**Table A1:** HEGY panel unit root outputs; * reject unit root presence at *p*-value = 0.05.

		HEGY Statistics					
Dependent Variable		lncal			Consumer Price Index (CPI)		
Class	Category	t1	t2	F34	t1	t2	F34
Carbohydrates	Cereals	−2.44	−2.58 *	7.3 *	1.39	0.06	1.68
	Water and non-alcoholic beverages	−2.13	−2.50 *	5.8 *	-	-	-
	Bread and bakery prod.	−2.50	−3.02 *	7.8 *	1.14	−1.12	3.16
	Cornmeal	−2.97 *	−3.12 *	8.1 *	2.76	2.10	0.24
	Flour	−3.80 *	−3.25 *	11.8 *	1.60	−1.20	0.15
	Fresh and canned vegetables	−2.79 *	−2.71 *	8.5 *	−2.82	−2.26	2.79
	Fruits	−3.13 *	−3.09 *	9.0 *	−1.19	−0.74	3.63
	Jam and compotes	−2.71 *	−2.96 *	7.4 *	2.88	−0.22	1.29
	Rice	-	-	-	1.39	0.06	1.68
	Potatoes	−2.12	−2.06	4.9	−3.16	−1.28	3.22
	Sugar products and sweets	−2.39	−2.72 *	5.6 *	3.34	2.84	4.86
	Sugar	−2.50	−2.91 *	8.5 *	2.41	1.31	7.46
Proteins	Meat and meat products	−1.80	−2.38 *	6.5 *	0.36	−0.48	1.26
	Milk	−4.96 *	−3.34 *	11.0 *	−2.01	−1.31	2.29
	Eggs	−3.18 *	−2.30 *	9.4 *	−1.16	−2.06	1.48
Fats	Cheese and cream	−2.57 *	−2.19 *	5.4	1.55	1.67	0.87
	Fats	−25.6 *	−12.9 *	195.7 *	−4.84	−5.41	1.29
	Sunflower, corn, and soybean oils	−2.96 *	−3.35 *	11.5 *	−4.85	−5.41	1.29
Variable		HEGY Statistics					
		t1	t2	F34			
Income		−2.78 *	−1.78	7.93 *			
UR		−3.65 *	−2.37 *	5.23			
Derived critical values at *p*-value = 0.05		−2.52	−2.07	5.47			

Model: constant + trend + seasonal dummies.
